# Supplementary material for: Factors associated with breast lesions among women attending select teaching and referral health facilities in Kenya: A cross-sectional study
Source: PLoS One. 2025 Jun 5;20(6):e0309182. doi: 10.1371/journal.pone.0309182 (PMC12140656; doi:10.1371/journal.pone.0309182)
Supplement: S1 File — (DOCX) [file pone.0309182.s001.docx]

# **Supporting information**

**S1 File:** Harvard Dataverse: Replication data for: Factors associated with breast lesions among women attending select teaching and referral health facilities in Kenya: a cross-sectional study, <https://doi.org/10.7910/DVN/2OXWTA>.
