# Supplementary material for: Factors associated with breast lesions among women attending select teaching and referral health facilities in Kenya: A cross-sectional study
Source: PLoS One. 2025 Jun 5;20(6):e0309182. doi: 10.1371/journal.pone.0309182 (PMC12140656; doi:10.1371/journal.pone.0309182)
Supplement: Attachment — Submitted filename: review report PONE-D-24-31297.pdf [file pone.0309182.s002.pdf]

The manuscript presents an interesting topic regarding the factors associated with breast lesions. However, the writing throughout the manuscript needs to be rechecked and improved.

Specific Comments:

Major Comments:

-Improve the English writing.

-Re-check and reformat the references.

-The discussion needs to be rewritten; it is almost entirely related to breast cancer. The author mentions in the title, abstract, and introduction a focus on breast lesions rather than breast cancer.

Minor Comments:

Lines 27-29: This information should be in the methods section of the manuscript, not in the methods part of the abstract.

Line 47: The terms "transitioned" and "transitioning countries" are not particularly meaningful; consider using alternative words.

Lines 47-48: The information is not found in the cited reference. Additionally, avoid mentioning "black" or "white" people without specifying racial or ethnic categories or populations.

Line 48: Instead of “unlike,” consider using a different word.

Line 51: This statement is too general and requires more detail: “About half of breast cancers can be explained by known risk factors (such as age and female gender), while the other half may remain unknown [5]. However, even with known risk factors, it is not certain that females with other risk factors will develop breast cancer.” Additionally, when referring to females, it is unnecessary to include "female gender."

Lines 117-134: This section should be cited only; all information is already present in the references, or it should be paraphrased.

Line 203: The introduction of Table 6.3 appears abruptly.

Line 203: The statement "$p < 0.2$" is mentioned, yet the author refers to it as significant. This needs to be rechecked in the text.
