# Supplementary material for: Factors associated with breast lesions among women attending select teaching and referral health facilities in Kenya: A cross-sectional study
Source: PLoS One. 2025 Jun 5;20(6):e0309182. doi: 10.1371/journal.pone.0309182 (PMC12140656; doi:10.1371/journal.pone.0309182)
Supplement: Attachment — Submitted filename: Response to Reviewers_24012025.docx [file pone.0309182.s004.docx]

Dr. Josephine Nyabeta Rioki

Faculty of Health Sciences

Department of Human Pathology

University of Nairobi,

Kenya

24 January 2025.

Email: jrioki@uonbi.ac.ke

Dear reviewers,

**PONE-D-24-31297: Factors associated with breast lesions among women attending select teaching and referral health facilities in Kenya: A cross-sectional study**

**_____________________________________________________________________________**

We thank you for taking time to review our manuscript in consideration for publication in your Journal. We are grateful for your observations and suggestions. Your comments were addressed as follows:

***Journal Requirements:***

***Comments 1:***

*Please ensure that your manuscript meets PLOS ONE's style requirements, including those for file naming. The PLOS ONE style templates can be found at*

[*https://journals.plos.org/plosone/s/file?id=wjVg/PLOSOne_formatting_sample_main_body.pdf*](https://journals.plos.org/plosone/s/file?id=wjVg/PLOSOne_formatting_sample_main_body.pdf) *and*

[*https://journals.plos.org/plosone/s/file?id=ba62/PLOSOne_formatting_sample_title_authors_affiliations.pdf*](https://c05y1x9s.r.us-east-2.awstrack.me/L0/https:%2F%2Fjournals.plos.org%2Fplosone%2Fs%2Ffile%3Fid=ba62%2FPLOSOne_formatting_sample_title_authors_affiliations.pdf/1/010f01925691277f-8b504255-ced6-4e04-aa69-6907f5bf81d1-000000/zROaB4zSnX6zd6zqD60v0c2-6Gg=179)

***Response:***

PLOS ONE’S style requirements have been considered.

***Comments 2:***

*Thank you for stating the following financial disclosure:“This research was supported by a grant from National Research Fund, Kenya (Postgraduate grant 2016) to JNR. The funder did not participate in the conduct of the research.” Please state what role the funders took in the study.  If the funders had no role, please state: "The funders had no role in study design, data collection and analysis, decision to publish, or preparation of the manuscript."*

***Response:***

The funders had no role in study design, data collection and analysis, decision to publish, or preparation of the manuscript.

***Comments 3:***

*Your ethics statement should only appear in the Methods section of your manuscript. If your ethics statement is written in any section besides the Methods, please delete it from any other section****.***

***Response:***

Ethics statemnent has been deleted from the abstract and it is only appearing in materials and methods section

***COMMENTS BY REVIEWER 1***

***Comment 1:*** *This case series study of 651 breast lump cases from two hospitals in Kenya. It may have its own value that it is a epidemiologic study from a country with limited resources. However, the association between lifestyle risk factors with breast lesions was studied in many previous studies, and the number of cases isn’t sufficient to support the study power in multinomial logistic regression.*

**Response:**
Even though the risk factors for breast lesions have been widely studied, there still remains a dearth of information on the factors that are associated with breast lesions among Sub-Saharan African women (Brinton et al. 2014). Understanding this set of factors may be key to tailoring interventions specific for this population. On sample size, Schwab (2002) contends that for a mutinomial logistic regression, a minimum of 10 cases per independent variable are necessary to sufficiently power it. With 12 predictors in our analysis, this translates to a sample of 120. Thus, our sample of 650 individuals adequately fulfils this requirement.

***Comment 2:*** *Cases included several diagnosis and the authors categorized the cases in four groups for statistical analysis. Therefore, although the authors underlined they included several types of abnormal cases of the breast, the number of cases was not enough to verify the association of the risk factors and the diseases.*

*I would recommend rather to divide the cases to two groups to non-benign cases and benign cases in a study of 651 cases in total.*

**Response:**
Regrouping the outcome into a binary variable as suggested by the reviewer would result in loss of vital statistical information considering that the studied breast lesion categories hold distinct cytopathological definitions. Moreover, as argued in the previous comment, the sample of 651 cases sufficiently powers the multinomial logistic regression analysis performed in the study.

***Comment 3:*** *In the body of the manuscript, the description are in detail in some parts but some important description needs be added or improved. For example, I think description of the rationale of the hypothesis of this study needs to be strengthened, and introduction of the medical system in Kenya needs explanation. For example, not many people outside Kenya are familiar with ‘Level 6’.*

**Response:** Improvement and addition of the description has been made in the manuscript (see page 4 and page 5 on the study sites )

In Kenya, we have referral and teaching health facilities/hospitals where specialized curative and diagnostics services are offered. Patients with breast masses for example are referred there from other health facilities in the country that do not have specialized human resource and equipment to render appropriate diagnoses.

***Comment 4:*** *In the methods, some parts - such as inclusion criteria and exclusion criteria - needs to be described in detail.*

**Response:** Revision has been made (see page 6)

***Comment 5:*** *In discussion, a detail discussion regarding study results could be summarized, and strengths and limitation of the study need to be added.*

**Response:** The discussion has been revised, and the strengths and study limitations have been included in the manuscript

***Comment 6:*** *In describing the results, the association found in this study could not be suggested as predictors of the outcomes since the study design was retrospective. In presenting odds ratios and confidence intervals in the tables, presenting number of cases and percentage in each cell together would help the authors to understand the main results.*

**Response:**

The term ‘predictors’ has been changed to ‘factors’ throughout the manuscript as suggested. However, even though it would have been ideal to present numbers of cases and percentages for the cells in the univariable analysis, this is likely to crowd out the main results from the table. More so, in the multivariable table, since the odds ratios are already adjusted for confounding, adding numbers of cases and percentages would be statistically erroneous.

***Comment 7:***

*I’d prefer to omit Table 1 or send it to supplements.*

**Response:**

The STROBE guidelines for reporting observational studies (Von Elm et al. 2008) dictate that for cross-sectional studies ‘details of methods of assessment/measurement for each variable of interest’ should be captured in the methodology section. This promotes transparency and assures reproducibility of the methods. Therefore, the suggestion to transfer this information to the supplement section would undermine this guideline.

***COMMENTS BY REVIEWER 2***

*Thanks for the chance to review your work. This is a cross-sectional study on breast lesions among women attending select teaching and referral health facilities in Kenya. I am conscious that this study is a clinical paper based on clinical data gained from real-world practice.*

***Comment 1:*** *Thus, it is important to reach a reliable conclusion depending on reasonable statistical analysis. I recommend that a statistic expert should comprehensively evaluate the methodology aspects.*

**Response:**

One of the co-authors (Dr. Mweu) is an epidemiologist/biostatistician and was involved in drafting/reviewing the methodological aspects of the study (particularly the statistical section) as well as the results portion of the manuscript. Consequently, some necessary revisions have been effected.

***Comment 2:*** *Please clarify the inclusion and exclusion criteria.*

**Response:** Revision has been made to the inclusion and exclusion criteria (see page 6)

***Comment 3:*** *Please consider to further discuss the potential risk factors for breast diseases in Kenya. What are the common ones that are shared among the world? What are the special ones limited to Kenya?*

**Response:**

An additional paragraph highlighting potential factors that could influence breast disease risk has been included (see page 16).

***Comment 4:*** *The participants in this study were women attending select teaching and referral health facilities. Why you chose this kind of sample? Were they representative enough?*

**Response:** We chose women attending teaching and referral health facilities for this study because these institutions are typically well-equipped to handle diagnostic and treatment services for breast lesions. These facilities attract patients from diverse socio-demographic and geographic backgrounds, making them a strategic choice for capturing a wide range of cases.

While the sample was limited to women attending these facilities, the selection aimed to include a population reflective of those seeking medical care for breast-related issues in Kenya. However, we acknowledge potential limitations in generalizability, particularly to women who may not have access to healthcare services. This limitation is discussed in the manuscript under the "Study Limitations" section **(**page 17).

***COMMENTS BY REVIEWER 3***

*The manuscript presents an interesting topic regarding the factors associated with breast lesions. However, the writing throughout the manuscript needs to be rechecked and improved.*

***Major Comments:*** *Improve the English writing.*

**Response:** English has been improved throughout the manuscript

***Comment:*** *Re-check and reformat the references.*

**Response:** Reformatting of the references has been done as per PLOS ONE requirements

***Comment:****The discussion needs to be rewritten; it is almost entirely related to breast cancer. The author mentions in the title, abstract, and introduction a focus on breast lesions rather than breast cancer.*

**Response:**
The discussion has been revised extensively to address this comment.
***Comment:*** *Lines 27-29: This information should be in the methods section of the manuscript, not in the methods part of the abstract.*

**Response**: The information in Lines 27-29 has been deleted from the manuscript

***Comment:*** *Line 47: The terms "transitioned" and "transitioning countries" are not particularly meaningful; consider using alternative words.*

**Response:** Paraphrasing of the paragraph has been done

***Comment:*** *Lines 47-48: The information is not found in the cited reference. Additionally, avoid mentioning "black" or "white" people without specifying racial or ethnic categories or populations.*

**Response:** The description of the population has been revised as advised. The correct reference has been included

***Comment:*** *Line 48: Instead of “unlike,” consider using a different word.*

**Response:** The whole sentence was revised.

***Comment:*** *Line 51: This statement is too general and requires more detail: “About half of breast cancers can be explained by known risk factors (such as age and female gender), while the other half may remain unknown [5]. However, even with known risk factors, it is not certain that females with other risk factors will develop breast cancer.” Additionally, when referring to females, it is unnecessary to include "female gender."*

**Response:** Revision have been made

***Comment:*** *Lines 117-134: This section should be cited only; all information is already present in the references, or it should be paraphrased.*

**Response:** Most of the content was deleted and citations made for the remaining information.

***Comment:*** *Line 203: The introduction of Table 6.3 appears abruptly.*

**Response:** Relabeling of the table has been made (see page11)

***Comment:*** *Line 203: The statement "p < 0.2" is mentioned, yet the author refers to it as significant. This needs to be rechecked in the text.*

**Response:**

Significance at the univariable analysis stage was gauged at a liberal $P$-value of ≤0.20 that aims to allow variables that are potentially negatively confounded have the opportunity to ‘express themselves’ in a multivariable analysis (Dohoo et al. 2012). Hence the term ‘significant’ here refers to the 20% cut-off level.

Thank you for the comments and suggestions by the reviewers, they have all been addressed as above.

I am looking forward to hearing from you.

References

Brinton, L.A., Figueroa, J.D., Awuah, B., Yarney, J., Wiafe, S, Wood, S, Ansong, D., Nyarko, K, Wiaffe-Addai, B., Clegg-Lamptey, J.N. 2014. Breast Cancer in Sub-Saharan Africa: Opportunities for Prevention. Breast Cancer Res. Treat., 144 (3): 467-478

Dohoo I., Martin, W., Stryhn, H. 2012. Methods in Epidemiologic Research, VER Inc. 1^st^ Edition, Prince Edward Island, Canada.

Schwab, J.A. 2002. Multinomial Logistic Regression: Basic Relationships and Complete Problems. University of Texas, Austin, Texas

Von Elm E, Altman DG, Egger M, Pocock SJ, Gøtzsche PC, Vandenbroucke JP. The Strengthening the Reporting of Observational Studies in Epidemiology (STROBE) statement: guidelines for reporting observational studies. Journal of Clinical Epidemiology [Internet]. 2008 Apr [cited 2024 May 31];61(4):344–9.

*Yours faithfully,*

*Dr. Josephine N. Rioki*
