# Supplementary material for: Factors associated with breast lesions among women attending select teaching and referral health facilities in Kenya: A cross-sectional study
Source: PLoS One. 2025 Jun 5;20(6):e0309182. doi: 10.1371/journal.pone.0309182 (PMC12140656; doi:10.1371/journal.pone.0309182)
Supplement: Attachment — Submitted filename: Response letter_2_JR_MM .docx [file pone.0309182.s005.docx]

Dr. Josephine Nyabeta Rioki

Faculty of Health Sciences

Department of Human Pathology

University of Nairobi,

Kenya

12 March 2025.

Email: jrioki@uonbi.ac.ke

Dear reviewers,

**PONE-D-24-31297R: Factors associated with breast lesions among women attending select teaching and referral health facilities in Kenya: A cross-sectional study**

**_____________________________________________________________________________**

We thank you for taking time to review our manuscript in consideration for publication in the PLoS ONE Journal. We are grateful for your observations and suggestions. Your comments were addressed as follows:

***COMMENTS BY REVIEWER 1***

***Comment 1:*** *I believe the improvements in the background and discussion enhance the readers’ understanding of the study’s objectives and results more clearly. I appreciate the authors’ efforts in collecting data from a low-resource setting and sincerely hope to see the study develop further.
However, the current study has some limitations regarding the available variables. Specifically, dose information is not provided, and most variables are categorized as binary (yes or no).*

**Response:**
On dose response and categorisation of variables, we firstly take note that this is a new comment raised by the reviewer (this had not been pointed out in the earlier feedback). Nonetheless, we agree with the reviewer’s assertion that capturing variables in a dose-response fashion would perhaps afford more useful statistical information than representing them binarily. However, for some variables e.g. smoking and alcohol histories, these practices are culturally frowned upon among women in many African rural populations and as such, it is typical to see studies dichotomising these variables. It is in this light that we recorded them as binary. Moreover, other few variables (as mentioned in the methods section) were recategorised owing to the small number of observations in some categories.

***Comment 2:*** *Additionally, important clinical characteristics, such as receptor status are unavailable. As a result, the study’s implications are somewhat limited in contributing novel insights beyond what is already known about breast cancer and benign lesions.*

**Response:**
Again, we note that this is a new issue raised by the reviewer. We nevertheless appreciate that capturing and subsequently assessing this variable could potentially yield some vital statistical information. However, it is worthwhile pointing out that, conventionally in our facilities, patients are only screened for the receptor status (ER, PR and HER2) once they have been diagnosed with breast cancer (malignancy) so as to guide effective therapy. Consequently, this status would only be expected for those women diagnosed with malignant breast lesions. More so, considering the observational design of this study, we could not retrieve this receptor status for a majority of these malignant-lesion patients. Notwithstanding, we are persuaded that this study sheds light on factors associated with breast lesions amongst women in Sub-Saharan Africa – evidence that is glaringly scarce.

***Comment 3:*** *The authors argue that the number of cases is sufficient for a multinomial logistic model. However, based on the large confidence intervals observed in several cells, it appears that some cells may have a small number of cases, which could lead to debatable conclusions.*

**Response:** As contended in our previous rebuttal letter, the sample available for this study was sufficiently sized to power the multinomial logistic regression. More importantly, the decision as to which which statistical model framework to adopt (multinomial vs binary logistic regression) rested on a delicate choice between (1) retaining universally accepted, standard cytopathological definitions of breast lesions (as per the International Academy of Cytology Yokohama System) and thus preserving critical statistical information inherent in the four breast lesion categories (multinomial logistic regression), and (2) upholding the statistical principle of parsimony (binary logistic regression being the simpler model). Considering that the patients in this study were independently classified into one of four distinctive breast lesion categories, with a view to retaining this useful information whilst keeping meaningful biological interpretability of the results, the multinomial logistic regression model framework was deemed an obvious choice. Albeit with a slight statistical shortcoming – wide confidence intervals for few variables. Despite this, statistically, the point estimates (odds ratios) should not be affected – thus the drawn conclusions should remain unchanged.

***Comment 4:*** *Nevertheless, I hope the authors successfully find a suitable platform to share this valuable dataset.*

**Response:** The dataset for this study is already deposited in a public repository: <https://doi.org/10.7910/DVN/2OXWTA>

***COMMENTS BY REVIEWER 2***

*The revised version addressed all previous comments and following are minor comments:
Line 325-326: need references
Line 363-364: need references*

**Response:**

A reference has been added in line 238-239 that necessitated a reference (see page 14). Moreso,

a statement that required a reference has been expunged since it was a general statement (see page 15).

We thank you for your thoughtful feedback.

*Yours faithfully,*

*Dr. Josephine N. Rioki*
