# Supplementary material for: Factors associated with breast lesions among women attending select teaching and referral health facilities in Kenya: A cross-sectional study
Source: PLoS One. 2025 Jun 5;20(6):e0309182. doi: 10.1371/journal.pone.0309182 (PMC12140656; doi:10.1371/journal.pone.0309182)
Supplement: Attachment — Submitted filename: Response letter_3_JR_MM_25_04_2025.docx [file pone.0309182.s006.docx]

Dr. Josephine Nyabeta Rioki

Faculty of Health Sciences

Department of Human Pathology

University of Nairobi,

Kenya

25 April 2025.

Email: jrioki@uonbi.ac.ke

Dear reviewers,

**PONE-D-24-31297R2: Factors associated with breast lesions among women attending select teaching and referral health facilities in Kenya: A cross-sectional study**

**_____________________________________________________________________________**

We thank you for taking time to review our manuscript in consideration for publication in the PLoS ONE Journal. We are grateful for your observations and suggestions. Your comments were addressed as follows:

***COMMENTS BY REVIEWER 3***

*Thanks to authors. All comments have been addressed.
The manuscript bring up an interesting study related to the factors associated with breast lesions among women attending select teaching and referral health facilities in Kenya.*

**Response:**
We appreciate the reveiwer’s sentiments.

***COMMENTS BY REVIEWER 4***

*It is illogical to combine etiologic risk factors research for benign and malignant lesions, just because the lesions occur within the same organ. This explains the contrarian results obtained by the researchers.
Most breast lesions present in specific age periods, e.g. fibroadenomas - the commonest breast lump occurs in early adulthood while fibroadenosis occurs perimenopausally. While the latter is associated with nulliparity and low parity, the former is not. The association of breast cancer with age, parity, breast feeding and menopausal status are well known.*

**Response:**

Actually, in concurrence with the reviewer’s assertion that the factors studied may have distinct effects depending on the lesion(s) under investigation, a multinomial logistic regression framework was employed since it permits the estimation of separate effects (odds ratios) of risk factors for each class of lesions. Moreover, considering the cross-sectional nature of the study and that study participants were cytologically classified into one of four possible lesion categories, it was deemed statistically sensible to assess the factor-lesion associations in a combined multinomial fashion. Furthermore, even though a number of these factors have been studied in other population settings, there is scarcity of evidence of factors associated with breast lesions amongst women in Sub-Saharan Africa.

***COMMENTS BY REVIEWER 5***

*This manuscript investigates factors associated with breast lesions among women attending referral facilities in Kenya. It contributes valuable data from a low-resource setting and addresses an understudied area, particularly in Sub-Saharan Africa. The study design is appropriate, the statistical methodology is generally sound, and the authors have made significant improvements across three rounds of revision.
The authors have adequately addressed the previous comments, including clarifying the sample size justification, improving methodological transparency, enhancing the discussion section, and revising the language and structure of the manuscript. Although certain limitations remain (e.g., binary categorization of variables and lack of receptor status data), the authors have provided reasonable justifications for these constraints.
I recommend acceptance after minor revisions, primarily to enhance clarity on limitations and ensure consistency in presentation.
My comments for the author:
1 .Please consider briefly reiterating the limitations related to binary classification of variables (e.g., smoking, alcohol use) in the discussion section to enhance transparency.
2. Please clarify whether the classification of breast lesions was based on cytology, histopathology, or imaging, and whether standardized diagnostic criteria were applied consistently across study sites. The potential for interobserver variability or misclassification bias should be addressed.
3. The study assesses parity and contraceptive use as binary exposures, but does not account for timing or duration. For example, recent childbirth or long-term contraceptive use may have different biological impacts than remote exposures. Please discuss how this limitation might influence the findings.*

**Response:**

We are appreciative of the positive feedback given by the reviewer. On point 1, the mentioned limitations have been added in the discussion segment – see lines 322-334. On point 2, the outcome definition has been refined and further information provided – see lines 126-129. On point 3, the impacts of recency of child birth and long-term contraceptive use have been explained – see lines 235-237 & 259-260.

***COMMENTS BY REVIEWER 6***

*This study looks at what causes breast lumps in Kenyan women by analyzing data from a cross-section of patients. It reveals some interesting connections between breast lumps and factors like how many children women have had, their exercise habits, and birth control use. What makes this research valuable is that it explores these health issues in a region where there hasn't been much research, helping us better understand what puts women at risk for both cancerous and non-cancerous breast problems.
Paper Weaknesses
1. Regarding reviewer 1, the author has not provided a convincing response. I recommend that the author increase the sample size, expand the analytical dimensions, update the statistical methods, and present the data more effectively.
2. The paper lacks detailed implementation guidelines, making it difficult for others to replicate the results. Providing additional details on hyperparameters, datasets, and code availability would enhance transparency.
3. How might the inclusion of receptor status data enhance the findings? Are there plans to incorporate this in subsequent research?
The paper should be submitted to another magazine with a lower impact factor.*

**Response:**

Point 1: As noted in our previous rebuttal, on sample size, Schwab (2002) contends that for a multinomial logistic regression, a minimum of 10 cases per independent variable is necessary to sufficiently power it. With 12 predictors in our analysis, this translates to a sample of 120. Thus, our sample of 651 individuals adequately fulfils this requirement. On ‘analytical dimensions’, ‘update the statistical methods’, and ‘present the data more effectively’, it is not clear what the reviewer specifically seeks to be addressed. Surprisingly, though, on the ‘comments to the author’ (point 3), the reviewer agrees that ‘the statistical analysis has been performed appropriately and rigorously’. Nevertheless, it is worth noting that we have endeavoured to strictly adhere to established guidelines for the reporting of cross-sectional studies to allow reproducibility of the study findings – see line 107.

Point 2: On ‘implementation guidelines’, it is unclear what the reviewer means here in reference to the study. Nonetheless, as aforementioned, the study has strictly complied to STROBE guidelines for reporting epidemiological studies to permit replicability of the results. On ‘hyperparameters’, these relate to the field of Bayesian statistics. It is important to note that our study applied a frequentist approach. It is apparent to the authors that some of the reviewer’s comments may not relate to the present study. On ‘datasets’, ‘code availability’, these had previously been deposited in a public data repository (Harvard Dataverse: <https://doi.org/10.7910/DVN/2OXWTA>) – but we have also included this information under the statistical section of the manuscript – see line 149. It is also worthwhile to note that on the ‘comments to the author’ (point 4), the reviewer agrees that ‘all data underlying the findings in the manuscript’ have been made available.

Point 3: On receptor status, this limitation has now been pointed out in the manuscript – see lines 328-334.

On whether the manuscript should be submitted to ‘another magazine’, we remain persuaded that our study conforms to the scope of PLOS One journal.

Reference:

Schwab, J.A. 2002. Multinomial Logistic Regression: Basic Relationships and Complete Problems. University of Texas, Austin, Texas

Again, we wish to thank the reviewers for their thoughtful feedback.

*Yours faithfully,*

*Dr. Josephine N. Rioki*
